# Cryopreservation of Red-Blood-Cell-Derived Extracellular Vesicles

**DOI:** 10.1021/acsomega.5c10099

**Published:** 2026-03-04

**Authors:** Kinga Ilyés, Tasvilla Sonallya, Judith Mihály, Anikó Gaál, Zoltán Varga

**Affiliations:** 1 Biological Nanochemistry Research Group, Institute of Materials and Environmental Chemistry, 280964 HUN-REN Research Centre for Natural Sciences, H-1117 Magyar tudósok körútja 2, Budapest, Hungary; 2 Faculty of Science, Hevesy György Doctoral School of Chemistry, Eötvös Loránd University, H-1117 Pázmány Péter sétány 1/A, Budapest, Hungary; 3 Department of Chemistry, Eszterházy Károly Catholic University, H-3300 Eszterházy tér 1, Eger, Hungary; 4 Department of Physical Chemistry and Materials Science, Faculty of Chemical Technology and Biotechnology, Budapest University of Technology and Economics, Műegyetem rkp. 3., H-1111 Budapest, Hungary; 5 Faculty of Medicine, Department of Biophysics and Radiation Biology - HUN-REN Office for Supported Research Groups, Semmelweis University, H-1094 Tűzoltó u. 37-47., Budapest, Hungary

## Abstract

Extracellular vesicles (EVs) are crucial in many physiological and pathological processes, and therefore, they are increasingly studied for their potential as diagnostic biomarkers, therapeutic agents, and drug carriers. Red-blood-cell-derived EVs (REVs) have gained particular interest due to their beneficial properties for drug delivery and their unique biophysical and molecular characteristics, which make them a practical model system for EV research. While EV cryopreservation methods have advanced in the past decade, REVs remain relatively understudied, with their hemoglobin-rich composition presenting unique storage challenges. To address this gap, we investigated how the vesicle concentration and buffer composition affected REV preservation to identify the best storage conditions. We evaluated changes in protein and lipid contents, hemoglobin retention, particle recovery, size distribution, and optical properties using a variety of analytical methods. Our results show that phosphate-buffered saline (PBS), despite its common use, leads to significant losses in vesicle number and major compositional changes. While human serum albumin (HSA) alone showed no protective effect, the combined action of HEPES, HSA, and trehalose (PBS-HAT) preserved the biochemical and biophysical integrity of REVs much more effectively. Furthermore, we found that the initial REV concentration at the time of freezing significantly affected particle recovery but not the composition of the EVs. Notably, a loss in particle concentration (30%) was already evident at an initial concentration of 1.2 × 10^11^ particles/mL. Our findings demonstrate that PBS-HAT offers an effective strategy for the short- to midterm cryopreservation of REVs above the initial concentration of 10^12^ particles/mL.

## Graphical abstract

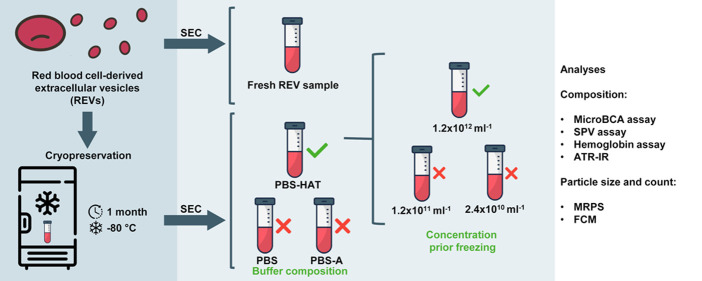

## 1 Introduction

Extracellular vesicles (EVs) are lipid-membrane-enclosed spherical particles released by most cells through exocytosis or cellular membrane budding mechanisms. Their size ranges from 30 nm to a few micrometers, and they contain diverse cargoes of biomolecules such as proteins, lipids, and nucleic acids (e.g., mRNA and microRNA). EVs are present in multiple biological fluids, reflecting the physiological or pathological state of their cell origin. Therefore, they are considered promising biomarkers. They can also transfer molecular information between cells across the extracellular space, which highlight their potential as drug carriers.^1,2^

The clinical use of EVs depends on effective preservation methods that protect their structural and functional integrity. Despite significant progress in recent years, long-term storage of EVs remains challenging and lacks standardization yet.^3–5^ The most commonly used approach is cryopreservation at – 80 °C, as recommended by the International Society for Extracellular Vesicles (ISEV) in 2013;^6^ however, more recent guidelines no longer provide a specific recommendation for storage conditions but emphasize the importance of clearly reporting the storage protocol used.^7^ The complex biomolecular composition of EVs creates high temperature dependence in their membrane fluidity,^8^ which makes them very sensitive to cryopreservation-related stresses. The formation of ice crystals together with osmotic imbalances and mechanical stress causes significant damage to lipid bilayers and internal cargo, which can compromise their structural integrity as well as biological functionality.^9^ These disruptive changes include vesicle aggregation, membrane rupture, decrease in the number of particles, and loss of useful biomolecular cargo, such as nucleic acids and surface proteins.^4,5,10–13^ To overcome current limitations and ensure the long-term stability of EVs for research and therapeutic use, it is crucial to optimize cryopreservation parameters, such as buffer composition or vesicle concentration, prior to freezing. Several studies have demonstrated that cryoprotectants and stabilizers can help preserve EV integrity during storage;^4,5,14^ however, the influence of the initial vesicle concentration prior to freezing remains a gap in the literature. Görgens et al. thoroughly investigated various cryopreservation conditions using HEK293T- and mesenchymal stem cells (MSCs)-derived EVs, identifying phosphate-buffered saline (PBS) supplemented with trehalose, human serum albumin (HSA), and HEPES as the most effective buffer.^4^ Although cell-derived EVs have a heterogeneous size distribution, the nanoparticle tracking analysis (NTA) technique used in their study cannot detect the full population of the EVs.

Among the different EV subtypes, red-blood-cell-derived extracellular vesicles (REVs) have attracted particular interest as drug carriers due to their beneficial characteristics. REVs offer a safe, scalable, and cost-effective platform for therapeutic applications since they can be produced easily in large quantities from red blood cell (RBC), the most abundant cell type in the body, collected routinely. Furthermore, they naturally lack nuclear and mitochondrial DNA and inherit their parent cells’ immunological compatibility. They have already been demonstrated to be effective carriers for RNA therapeutics in gene therapy^15^ and anticancer drugs, where their efficacy of specific delivery was further enhanced by conjugation of targeting ligands.^16^ REVs can be used as a model system for EV research since they have an almost spherical shape and a relatively monomodal size distribution within the larger 100–300 nm range,^17^ allowing detection of the full EV population. REVs are also distinct from other EV subtypes in terms of molecular composition. Hemoglobin is their most abundant protein cargo,^18^ which influences their light scattering and absorption.^19^

Despite significant progress in EV preservation reported in the literature, relatively little attention has been paid to REVs specifically. Their distinct composition presents unique challenges for long-term storage, as the loss or degradation of hemoglobin during freezing undermines the precision of optical detection methods, given that light scattering depends on the particle size, shape, and refractive index.^20^ Alterations in the REV composition may therefore result in inaccurate measurements of size, concentration, or surface marker expression.

The goal of this study is to develop a standardized cryopreservation protocol for REVs to maintain their structural and biochemical integrity successfully. We systematically evaluated how buffer composition and initial vesicle concentration influence the freezing process by assessing their effects on critical parameters including biochemical composition, hemoglobin retention, particle size, recovery rate, and biophysical stability. Our results provide new perspectives on how storage conditions impact REV quality and recovery to improve reliable preservation and accurate subsequent analysis to facilitate their wider use in research and therapeutic applications.

## 2 Materials and Methods

### 2.1 REV Isolation and Freezing

Ethical permission for using human blood samples was obtained from the Scientific and Research Ethics Committee of the Hungarian Medical Research Council (1966-6/2022/EÜIG). All procedures were conducted in accordance with the guidelines and regulations of the Declaration of Helsinki and its subsequent revisions.

Eighteen milliliters of fresh peripheral blood was collected from three healthy adult volunteers into 6 mL of K_3_EDTA tubes (Vacuette, Greiner Bio-One, Austria). RBCs were isolated by centrifugation at 800*g* for 10 min at 4 °C (Hermle Z 327 K, swing out rotor (221.71 V20) with adapters for 15 mL centrifuge tubes). After removal of the plasma and the buffy coat, the RBC pellet was washed three times with physiological saline solution (0.9% SALSOL, TEVA, Hungary) using the same centrifugation conditions to eliminate residual platelets and leukocytes. The final RBC pellet was resuspended in phosphate-buffered saline (PBS, Dulbecco’s PBS (10×), cat. PBS-10XA, Capricorn Scientific, diluted to 1×, pH 7.4, 0.2 μm filtered) and stored at 4 °C for 7 days to produce REVs.

Following 1 week of incubation, samples were subjected to two consecutive centrifugation steps to remove red blood cells and debris: first at 2500*g* for 10 min followed by 3000*g* for 15 min (Hermle Z 327 K, wing out rotor (221.71 V20) with adapters for 15 mL centrifuge tubes). The resulting supernatants were then centrifuged at 16,000*g* for 30 min at 4 °C (Eppendorf 5415R, F45-24-11 rotor) to pellet REVs. The pellets were resuspended in PBS, equally divided into three tubes, and centrifuged again at 16,000*g* for 30 min at 4 °C (Eppendorf 5415R, F45-24-11 rotor). The isolated EV samples were resuspended in the three different buffers: (A) PBS, (B) PBS-A (1× PBS supplemented with 0.2% human serum albumin (HSA, cat. A-1887 1G, Sigma-Aldrich)), and (C) PBS-HAT (1× PBS supplemented with 25 mM HEPES (cat. H4034-25G, Sigma-Aldrich), 0.2% human serum albumin (HSA, cat. A-1887 1G, Sigma-Aldrich), and 25 mM trehalose (d-(+)-trehalose dihydrate, cat. T0167-25G, Sigma-Aldrich)).^4^ All buffers were filtered through 0.22 μm filters before usage.

EV samples were aliquoted in concentrated form and, in the case of the PBS-HAT buffer, also in 10× and 50× diluted forms. Samples were either analyzed fresh or stored at −80 °C for 4 weeks following snap-freezing in liquid nitrogen.

To remove co-sedimented proteins and protein aggregates from fresh and thawed REVs, size exclusion chromatography (SEC) was performed using qEVsingle columns (IZON Science Ltd., New Zealand). According to the manufacturer’s protocol, 500 μL of EV suspension was loaded onto the column followed by elution with PBS. The initial 2.5 mL void volume was discarded, and 20 fractions of 500 μL each were collected. EV-containing fractions 2 and 3 were later pooled and concentrated by centrifugation (16,000*g*, 30 min, Eppendorf 5415R, F45-24-11 rotor). Purified REVs were finally recovered by resuspension of the pellet in 200 μL of PBS.

### 2.2 REV Characterization

#### 2.2.1 Freeze-Fracture Transmission Electron Microscopy (FF-TEM)

The morphology of freshly isolated, concentrated REVs was analyzed by FF-TEM to assess sample purity and provide visual confirmation of vesicle structures. Vesicle samples were preconcentrated by centrifugation (16,000*g*, 30 min, Eppendorf 5415R, F45-24-11 rotor) and then mixed with glycerol (Sigma-Aldrich) in a sample-to-glycerol volume ratio of 3:1 to prevent freezing-induced artifacts. Two microliters of the mixture was pipetted onto a gold sample holder and rapidly frozen by immersion into liquid Freon. Fracturing was carried out at −100 °C using a Balzers BAF 400D freeze-fracture system (Balzers AG, Liechtenstein). Platinum–carbon replicas were prepared by vacuum evaporation followed by washing with a surfactant-containing aqueous solution and rinsing with distilled water. The resulting replicas were transferred onto 200-mesh copper grids and examined using a MORGAGNI 268D transmission electron microscope (FEI, The Netherlands).

#### 2.2.2 Protein Quantification by the MicroBCA Assay

Protein quantification was performed using the Micro BCA Protein Assay Kit (Thermo Fisher Scientific, USA). A standard curve was prepared with bovine serum albumin (BSA, cat. no. A9418-10G, Sigma-Aldrich) at final concentrations (μg/mL) of 0, 5, 10, 25, 50, 75, 125, 250, 500, 750, 1000, and 1500. The 20 collected SEC fractions from both fresh and frozen samples stored in PBS, PBS-A, and PBS-HAT were analyzed according to the manufacturer’s instructions. Absorbance was measured at 562 nm in UV-transparent 96-well plates using a microplate reader (Bio-Tek Synergy 2 MultiMode Microplate Reader) operated by the Gen5 3.11 Software.

#### 2.2.3 Hemoglobin Content by UV–Vis Spectroscopy

Hemoglobin content in SEC fractions was quantified spectrophotometrically by measuring the absorbance of 75 μL of each sample at 414 nm in a UV-transparent 96-well plate by using a microplate reader (Bio-Tek Synergy 2 MultiMode Microplate Reader) operated by the Gen5 3.11 Software. Calibration was performed with lyophilized hemoglobin (cat. H7379, Sigma-Aldrich) at final concentrations (μg/mL) of 0, 25, 125, 250, 500, and 1000, and hemoglobin content was calculated from the standard curve.

#### 2.2.4 Lipid Quantification by the SPV Assay

Lipid quantification was performed for the pooled and concentrated REV samples using a colorimetric assay based on sulfuric acid and vanillin–phosphoric acid reaction (SPV assay).^21^ A 1 mg/mL DOPC liposome standard solution was prepared in PBS and used to generate a 2-fold serial dilution ranging from 16 to 0.25 μg in 40 μL final volume, with PBS as blank. For each standard and sample, 40 μL was mixed with 200 μL of 96% sulfuric acid and incubated at 90 °C for 20 min in a fume hood. After cooling at 4 °C for 5 min, 120 μL of vanillin reagent (prepared in 17% phosphoric acid) was added. The reaction mixture (280 μL) was transferred to a 96-well UV-transparent plate and incubated at 37 °C for 1 h. Absorbance was measured at 540 nm by using a microplate reader (Bio-Tek Synergy 2 MultiMode Microplate Reader) operated by the Gen5 3.11 Software. Lipid content was calculated from the standard curve.

#### 2.2.5 Attenuated Total Reflection Infrared Spectroscopy (ATR-IR)

ATR-IR spectra were recorded using a Varian 2000 FTIR Scimitar Series spectrometer (Varian Inc., USA) equipped with a liquid-nitrogen-cooled mercury–cadmium–telluride (MCT) detector and a single-reflection diamond ATR accessory (Specac Ltd., UK). Three microliters of each sample was applied to the ATR crystal and air-dried under nitrogen to form a thin film. Spectra were collected at room temperature by 64 scans at a 2 cm^–1^ resolution followed by ATR correction. Spectral processing was performed using the GRAMS/32 software (Galactic Inc., USA).

The total protein content was estimated from the intensity of the amide I band. The spectroscopic protein-to-lipid (P/L) ratio of REVs was calculated by integrating the areas of the amide I band and the C–H stretching region (3000–2800 cm^–1^) typical for phospholipid content. This ratio can be applied to distinguish vesicle subtypes, evaluate sample purity, and monitor if the composition of the sample changes during storage.^22^

#### 2.2.6 Flow Cytometry Analysis (FCM)

Flow cytometric analysis was performed on a CytoFLEX S flow cytometer (V4-B2-Y0-R3 configuration, Beckman Coulter, USA) operated with the CytExpert Software v2.5 (Beckman Coulter, USA). For single EV detection, the violet side scatter (VSSC-height) channel was used as the trigger, with the threshold manually set to 1500 arbitrary units. Detector gain was set to 250 for VSSC and 2000 for PE. Each sample was analyzed for 60 s at a flow rate of 10 μL/min.

Concentrated REV samples were diluted 2.5 × 10^4^-fold prior to measurement. In the case of samples that had already been prediluted 10× and 50× during preparation, the applied dilution was adjusted accordingly, resulting in 2.5 × 10^3^- and 5 × 10^2^-fold dilution, respectively. Each condition was analyzed in three independent replicates, and values are reported as the mean ± SD. EVs were labeled using a fluorochrome-conjugated antibody targeting glycophorin A, a membrane protein characteristic of red blood cells. Specifically, 25 μL of 3 × 10^11^ particles/mL REV suspension was incubated with 1 μL of PE-conjugated anti-CD235a antibody (0.2 mg/mL, cat. 12-9987-82, ThermoFisher Scientific, USA) for 30 min at 37 °C. The antibody concentration was adjusted to achieve the same ratio in the case of 10× and 50× diluted samples, resulting in adding 2 and 0.5 μL of 10× diluted CD235a-PE antibody, respectively.

Light scatter calibration was performed using silica nanoparticles of defined diameters (SNP088, SNP083, SNP082, and SNP073).^23^ Flow cytometry standard (FCS) files were calibrated with the FCMPASS software (v3.10; https://nano.ccr.cancer.gov/fcmpass) as described previously.^24,25^ Flow cytometry data were processed and analyzed using the Kaluza Analysis Software (version 2.1, Beckman Coulter, USA).

#### 2.2.7 Microfluidic Resistive Pulse Sensing (MRPS)

REV concentration and size distribution were determined in fresh and thawed samples stored in PBS, PBS-A, and PBS-HAT with microfluidic resistive pulse sensing (MRPS) by a Spectradyne nCS1 instrument (Spectradyne LLC, USA) based on detecting electrical resistance changes as particles pass through a nanoconstriction. Due to its low sample volume requirement and high sensitivity, this method also enabled the analysis of 10× and 50× diluted samples prior to freezing. Measurements were performed using C-400 cartridges suitable for particle sizes between 65 and 400 nm, with calibration based on PS90 polystyrene standards. Concentrated, 10×, and 50× REV samples were diluted 100×, 10×, and 2×, respectively, in 0.05% Poloxamer188-PBS nonionic surfactant (Gibco, USA),^26^ prefiltered using 100 kDa MWCO Amicon Ultra 0.5 mL filters (Merck Millipore, Germany). Data represent the mean ± standard deviation of three independent measurements per sample.

#### 2.2.8 Zeta Potential Measurement

Zeta potential (ζ potential) measurements were performed using a Zetasizer Nano ZS instrument (Malvern Panalytical GmbH, Germany) equipped with disposable folded capillary cells to assess the impact of different freezing conditions on the surface charge of REVs. The initial samples were prepared in PBS at 1× concentration containing 137 mM NaCl and 2.7 mM KCl at pH 7.4. Concentrated samples were diluted 50-fold with PBS, while 10× and 50× prediluted samples were used in 5× and without additional dilution, respectively. Prior to measurement, all samples were subsequently diluted 10-fold in 150 mM sucrose solution, resulting in a final sucrose solution with a PBS concentration of 0.05× (i.e., 142.5 mM sucrose, 6.85 mM NaCl, and 0.135 mM KCl). The sucrose solution was chosen to minimize the osmotic pressure, while the electrolyte content of the diluted PBS ensures adequate conductivity. Measurements were conducted at 25 °C after a 3 min equilibration period. Each measurement was repeated in triplicate, and the mean values and standard deviations were calculated.

### 2.3 Statistical Analysis

Statistical analyses were performed using one-way ANOVA followed by Bonferroni’s multiple comparison test in GraphPad Prism 6 (GraphPad Software, San Diego, USA).

## 3 Results

### 3.1 Characterization of Fresh REVs

Figure 1a shows the experimental workflow of the study. REVs obtained after centrifugation at 16,000*g* were resuspended in different buffer formulations (PBS, PBS-A, or PBS-HAT at various dilutions), stored at −80 °C for 4 weeks, and then thawed. Both fresh and thawed samples were purified by size-exclusion chromatography (SEC) and analyzed for composition (e.g., protein, lipid, and hemoglobin content) and particle characteristics (size and concentration) using microBCA, SPV, ATR-IR, MRPS, and flow cytometry.

**Figure 1:**
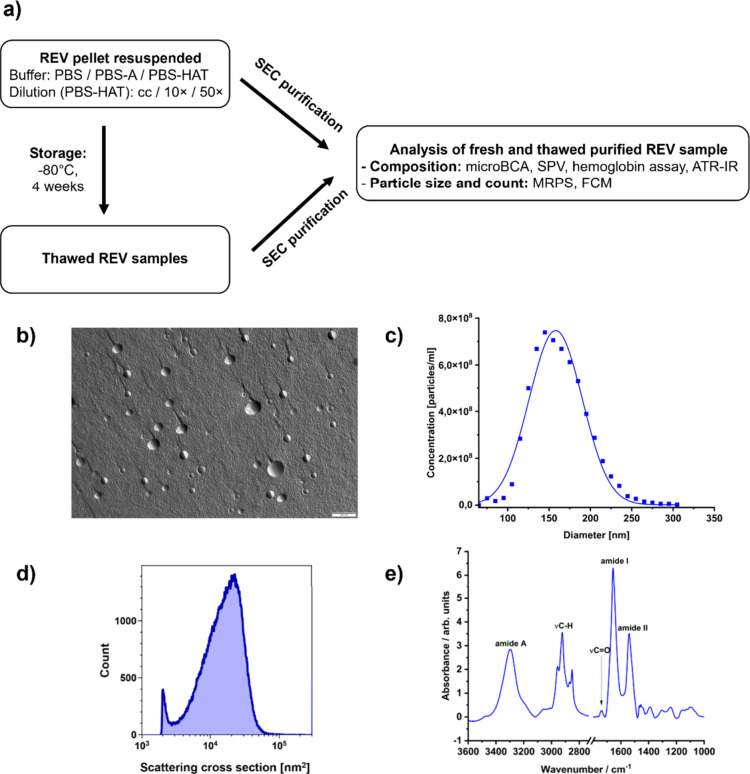
Schematic overview of the experimental workflow (a). Representative TEM image (b), MRPS size distribution (c), FCM scattering cross-section profile (d), and ATR-IR spectra (e) of freshly isolated REV sample.

Morphological characterization of the freshly prepared REV sample by FF-TEM (Figure 1b) showed predominantly spherical structures with diameters in the range of 100 to 300 nm, consistent with the expected size and shape of REVs.^22,27^ The surface of most vesicles appears textured, with granule-like features, which represent the native membrane-associated proteins.^28^ The size distribution and concentration of the vesicles were measured using MRPS (Figure 1c), showing a monodisperse profile that fits well to log-normal distribution, similarly to previous results,^29^ with a mean diameter of 156.6 ± 1.2 nm and concentration of (1.213 ± 0.178) × 10^12^ particles/mL over the size range from 65 to 400 nm. This was further confirmed by FCM results (Figure 1d). The composition and P/L ratio were characterized by ATR-IR (Figure 1e), showing a similar profile as in the literature.^22^

### 3.2 Compositional Changes in Different Storage Buffers upon Freezing

To investigate the impact of freezing on the molecular composition of REVs, fresh and thawed samples purified by SEC were compared following storage in three different buffer compositions.

Protein elution profiles across SEC fractions were analyzed using the microBCA protein assay (Figure 2a–c). The EV-enriched fractions correspond to fractions 2 and 3. In the fresh PBS-A and PBS-HAT samples, residual buffer-associated proteins were detected in fractions 9–19. Notable differences were observed between fresh and thawed samples stored in PBS and PBS-A, with thawed samples showing reduced protein content in EV-rich fractions (49% and 46% recovery rate, respectively) and increased levels of free proteins in later fractions. In contrast, the SEC profile of the samples stored in PBS-HAT remained almost exactly like that of the fresh sample, suggesting the improved preservation of vesicle-associated proteins.

**Figure 2:**
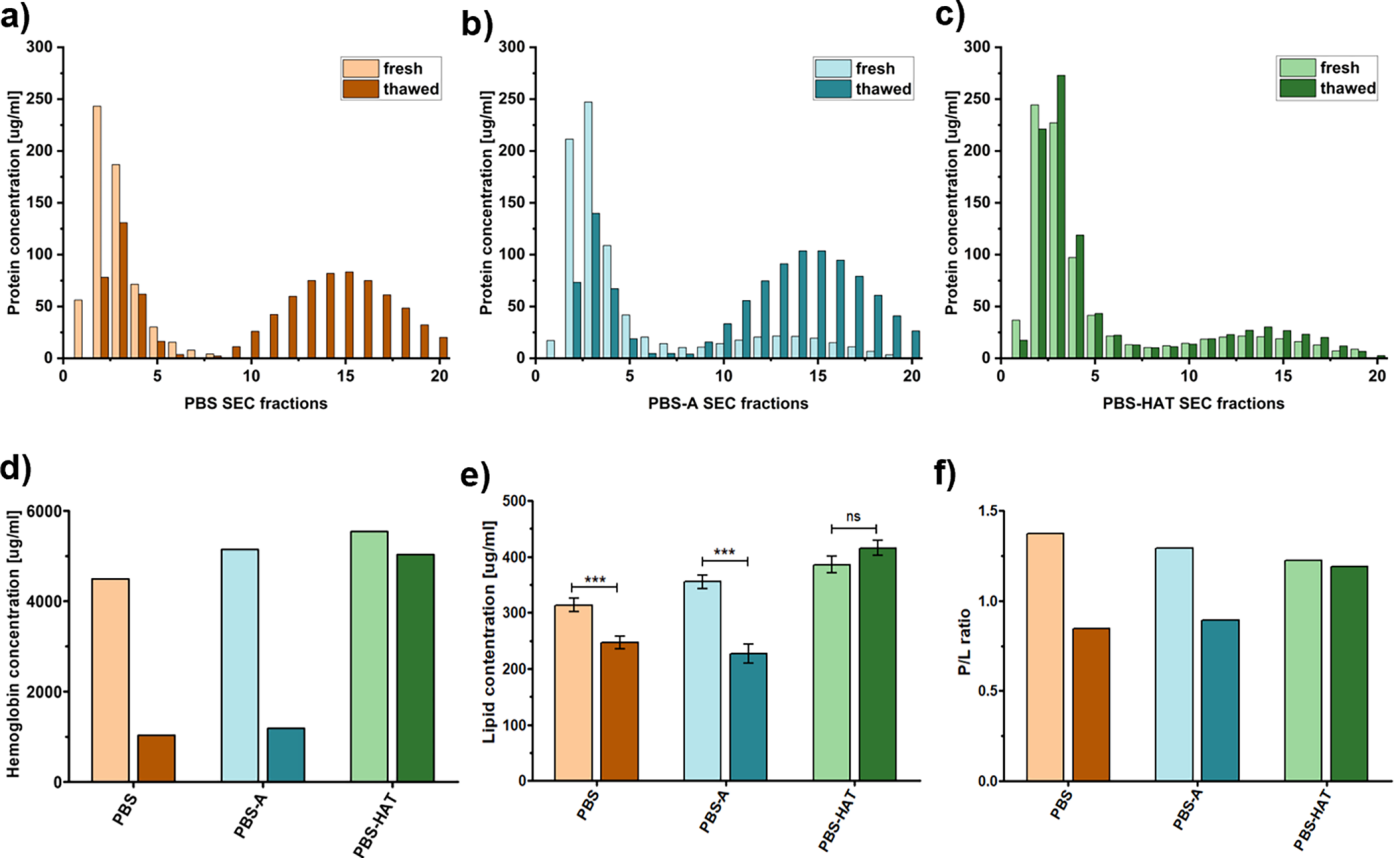
Protein concentration across SEC fractions (a–c), hemoglobin concentration (d), and lipid concentration (e) comparing fresh and thawed samples in PBS, PBS-A, and PBS-HAT buffers. The calculated protein-to-lipid (P/L) ratios based on colorimetric assay results are represented on the bar chart (f). Data for lipid concentration represent mean ± SD, and statistical significance was assessed by one-way ANOVA followed by Bonferroni’s multiple-comparison test.

Hemoglobin content was quantified by measuring absorbance at 414 nm after pooling and concentrating the EV-enriched fractions in both fresh and thawed samples (Figure 2d). PBS and PBS-A had a recovery rate of 23.0%, while PBS-HAT showed a significantly higher recovery rate of 90.8%.

Similarly, lipid concentration (Figure 2e) was significantly reduced in thawed PBS and PBS-A samples compared to fresh ones; however, the recovery rate is higher (78.7% and 64.0% respectively) than in the case of hemoglobin content. PBS-HAT preserved lipid content at levels not significantly different from fresh samples.

The protein-to-lipid (P/L) ratio (Figure 2f) was calculated based on the results of the microBCA protein and SPV lipid assay. The P/L ratio can be used as a qualitative marker for extracellular vesicle characterization, as it reflects vesicle integrity and purity.^30^ PBS-HAT samples showed the highest stability, with minimal changes in the P/L ratio between fresh and thawed states. In contrast, vesicles stored in PBS and PBS-A showed a decreased P/L ratio, indicating a greater loss of protein relative to lipid content in the absence of effective cryoprotectants.

### 3.3 Structural and Compositional Analysis by ATR-IR Spectroscopy

ATR-IR spectroscopy was used to evaluate the effect of freezing on the composition of the REVs. The spectra (Figure 3a–c) are dominated by protein-related amide bands—amide A, amide I and amide II—at approximately 3288, 1656, and 1540 cm^–1^, respectively. Characteristic sharp lipid bands can also be observed, including methylene asymmetric and symmetric stretching vibrations of the lipid acyl chains at ∼2924 and ∼2856 cm^–1^, respectively, as well as the ester carbonyl stretch of phospholipids, triglycerides, and cholesterol esters at ∼1734 cm^–1^.^31^

**Figure 3:**
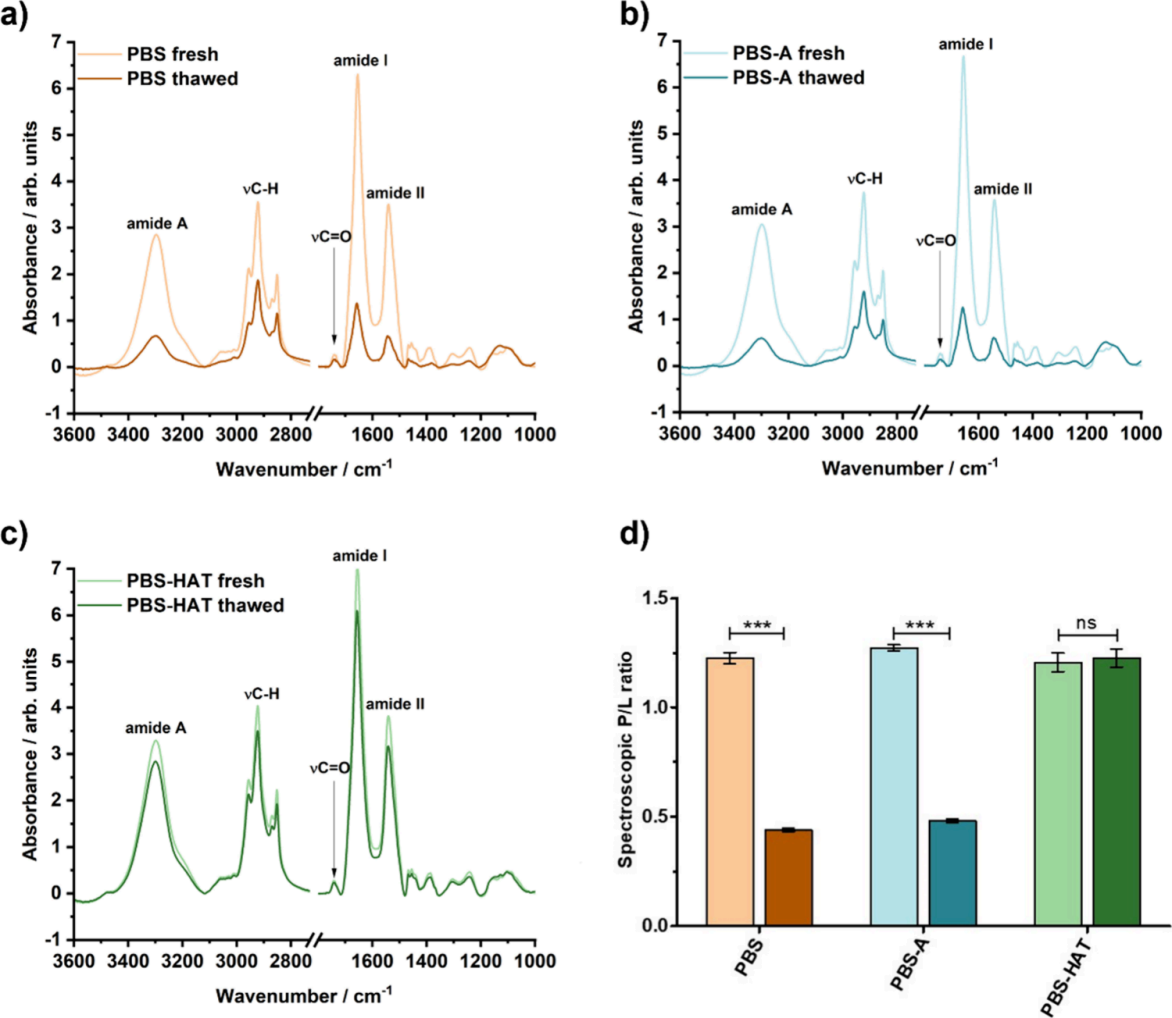
ATR-IR spectra comparing fresh and thawed samples in PBS, PBS-A, and PBS-HAT buffers (a–c) and calculated P/L ratios based on the spectra (d). Data represent mean ± SD. Statistical significance was assessed by one-way ANOVA followed by Bonferroni’s multiple-comparison test.

Comparison of fresh and thawed samples in different buffers (Figure 3a–c) revealed an evident decrease in both protein- and lipid-associated bands in samples frozen in PBS and PBS-A; however, the PBS-HAT samples showed no notable spectral changes. To quantify these differences, the spectroscopic protein-to-lipid (P/L) ratio was calculated by integrating the amide I region (1700–1600 cm^–1^) and the lipid-associated C–H stretching region (3000–2800 cm^–1^) (Figure 3d). Fresh samples showed a typical spectroscopic P/L ratio of approximately 1.2, consistent with literature values for REVs.^31^ This ratio remained the same in PBS-HAT samples following freezing, whereas in PBS and PBS-A buffers, the P/L ratio decreased significantly.

### 3.4 Analysis of Concentration and Size Distribution Changes Induced by Freezing in Different Buffers and Vesicle Concentrations

MRPS was employed to evaluate the effect of freezing on the size distribution and concentration of REVs in different buffers and different dilutions. The representative MRPS size distribution curves (Figure 4a–c) and the corresponding bar chart of mean diameters (Figure 4d) demonstrate that freezing caused slight changes in particle size for all buffer types. While samples stored in the PBS-HAT buffer at all concentrations showed a slight shift toward larger diameters, REVs frozen in PBS and PBS-A displayed a slight decrease in average size. However, all observed size changes remained within the range of 147 to 172 nm and cannot be considered statistically significant.

**Figure 4:**
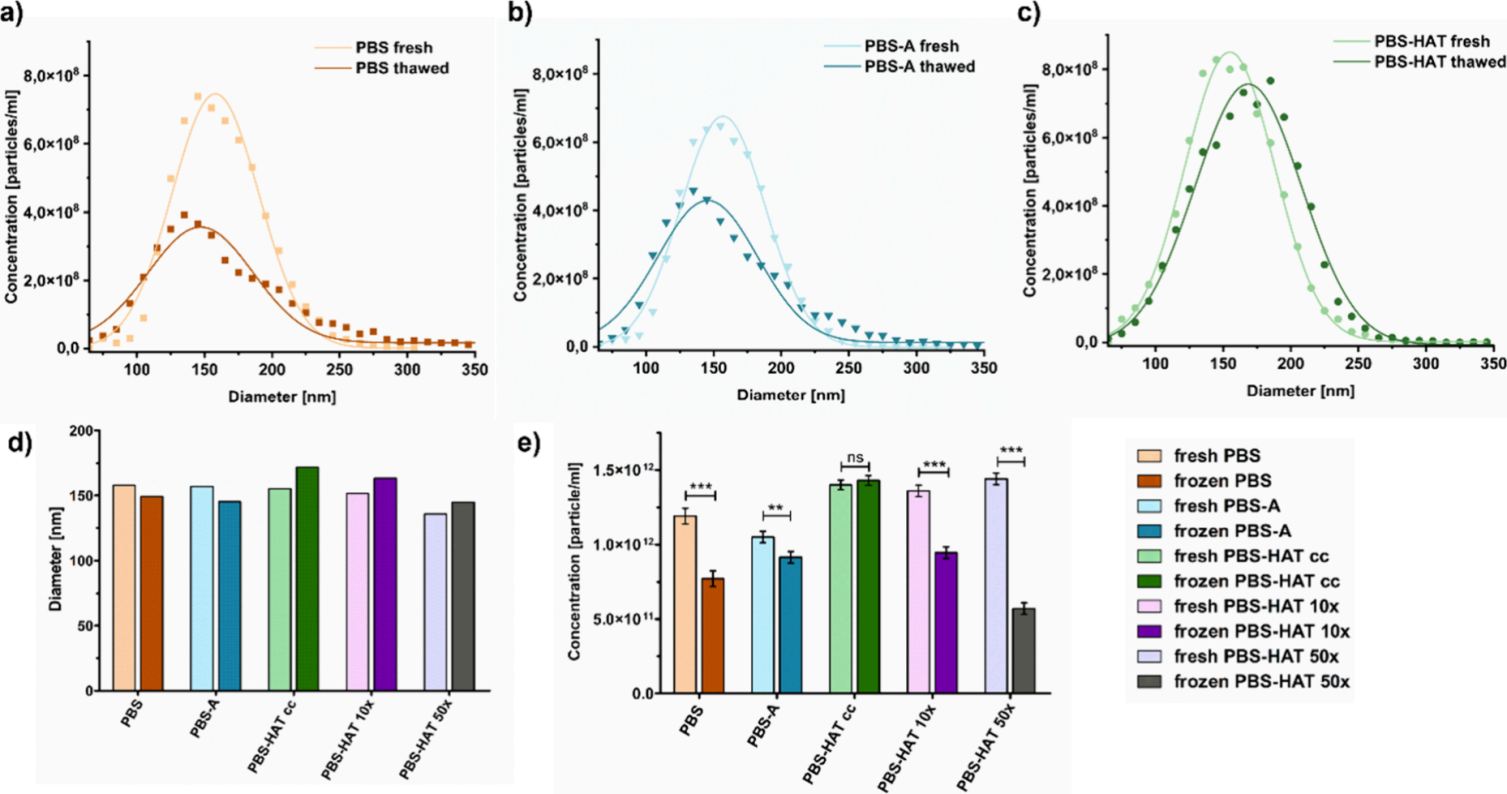
Size distribution profile of REVs (a–c), particle diameter (d), and particle concentration presented as a bar chart (e), measured by MRPS, comparing fresh and thawed samples in PBS, PBS-A, PBS-HAT cc, PBS-HAT 10×, and PBS-HAT 50×. Data represent mean ± SD. Statistical significance was assessed using one-way ANOVA followed by Bonferroni’s multiple-comparison test.

In contrast, the particle concentration (Figure 4e) was significantly affected by the freezing process. Samples stored in PBS and PBS-A showed a significant reduction, with recovery rates of 65% and 87%, respectively. The PBS-HAT buffer preserved particle counts more effectively, with no substantial loss in the concentrated (cc) form. However, when diluted 10× or 50× before freezing, even PBS-HAT samples showed notable concentration loss, 69% and 40% recovery, respectively.

FCM offers complementary information because it is more sensitive to changes in the refractive index and sample composition. This sensitivity makes it possible to identify structural or biochemical alterations in EVs that may result from freeze–thaw stress. The results show that in samples stored in PBS and PBS-A, a significant decrease in particle concentration was observed after thawing (Figure 5f), accompanied by a shift in the scattering cross-section distribution toward lower values (Figure 5a,b). In contrast, PBS-HAT samples showed no change in scattering cross-section distributions between fresh and thawed states (Figure 5c–e); however, particle concentration was still significantly reduced in diluted PBS-HAT samples upon thawing (Figure 5f).

**Figure 5:**
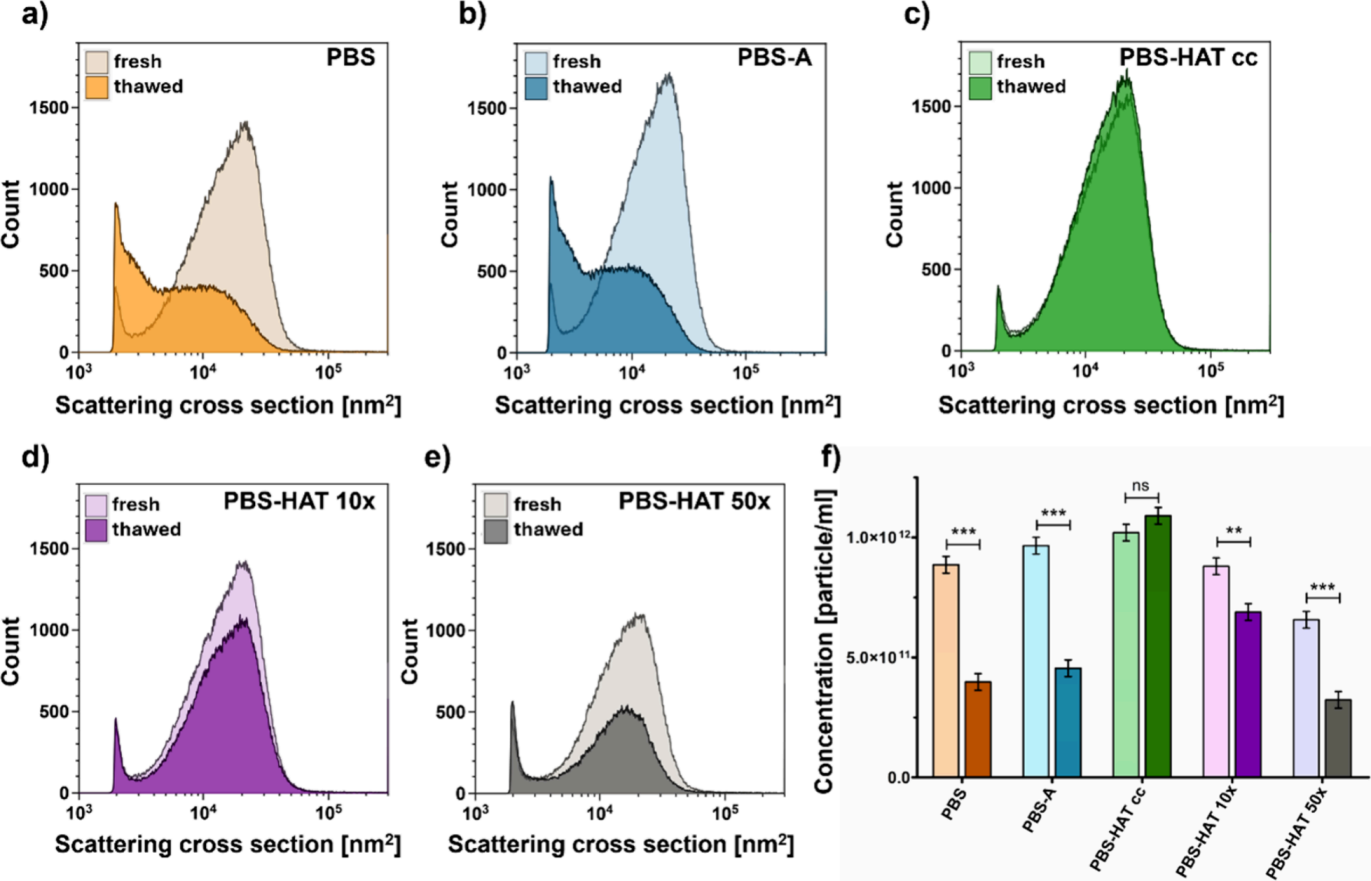
Scattering cross-section distribution profiles of REV samples measured by FCM under fresh and thawed conditions in different buffers: PBS, PBS-A, PBS-HAT cc, PBS-HAT 10×, and PBS-HAT 50× (a–e). Particle concentrations are presented as bar charts (f), comparing fresh and thawed samples across the conditions. Data represent mean ± SD. Statistical significance was assessed by one-way ANOVA followed by Bonferroni’s multiple-comparison test.

As REVs derive from the erythrocyte membrane, they retain membrane proteins of the parent cell, including CD235a (glycophorin A), the most abundant sialoglycoprotein of the human red blood cell membrane.^32,33^ Owing to its high abundance, membrane localization, and erythroid specificity, CD235a is widely applied for the identification and labeling of REVs. Fluorescence labeling with CD235a-PE antibody (Figure 6a,b) revealed the appearance of a new vesicle subpopulation following freezing in PBS and PBS-A, characterized by positive labeling for CD235a but reduced scattering cross-section (Figure 6c,d). In contrast, PBS-HAT preserved the native scattering and labeling profile in concentrated form (Figure 6e,f) but slightly increased the emergence of new populations in diluted 10× and 50× forms (Figure 6g).

**Figure 6:**
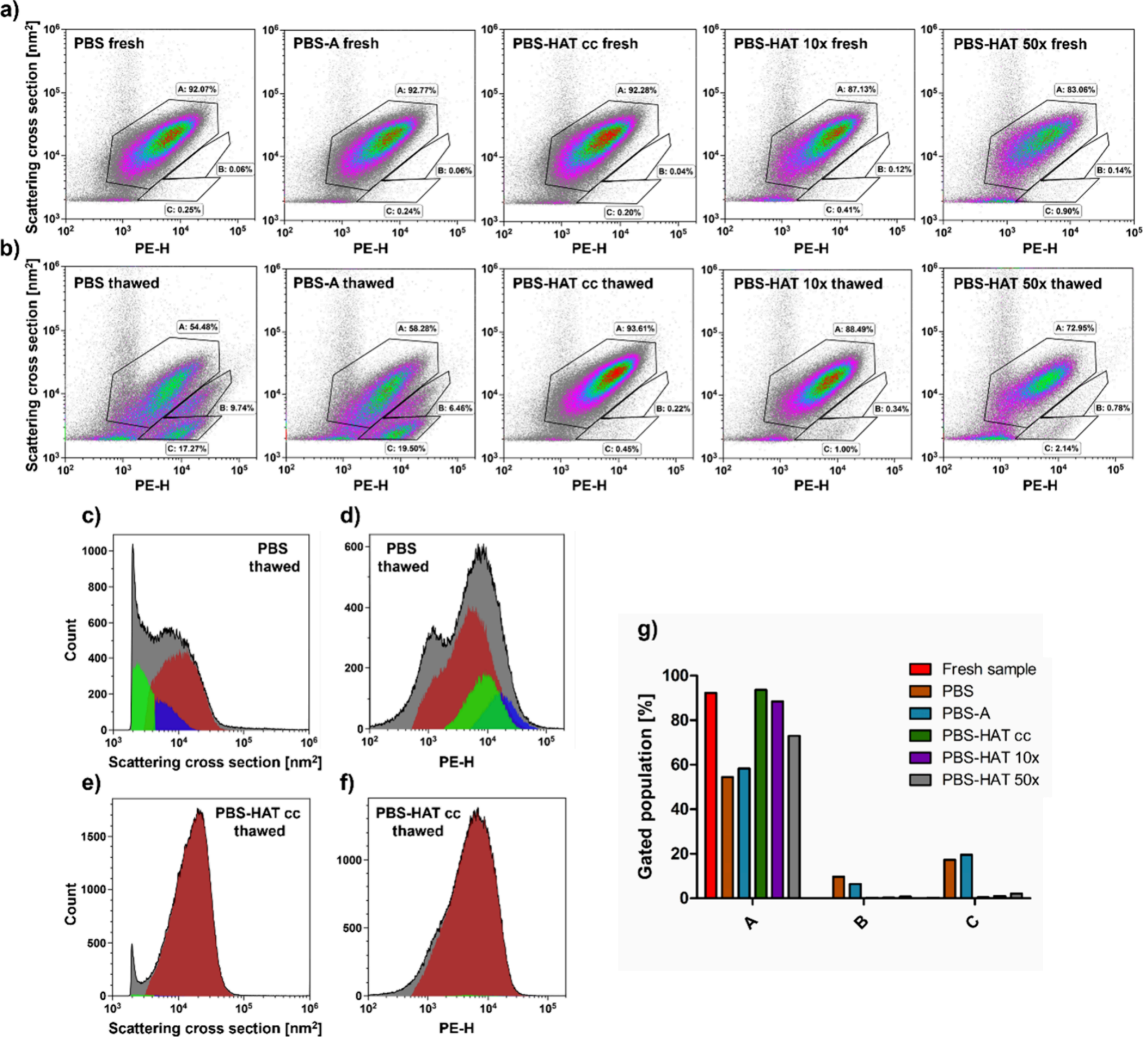
Flow cytometry analysis of particles labeled with CD235a-PE antibody in fresh (a) and thawed (b) samples under different buffer conditions (PBS, PBS-A, PBS-HAT, PBS-HAT 10×, and PBS-HAT 50×). Populations A–C were defined based on the scattering cross section and PE fluorescence intensity. Histograms with color-coded overlays for gates A (red), B (blue), and C (green) show the distribution of scattering (c, e) and fluorescence (d, f) signals in thawed samples in PBS and PBS-HAT, respectively. The relative proportions of events within each gate in thawed samples under all buffer conditions are summarized in the bar chart (g). Fresh sample data are colored red as a reference.

### 3.5 Zeta Potential Measurements

We measured the zeta potential before and after freezing to evaluate the effect of freezing under various buffer conditions on the surface charge of the REVs (Figure 7). While zeta potential values remained relatively stable in most conditions, a significant increase was observed in PBS buffer following freezing. However, overall changes were moderate, suggesting that surface charge changed only slightly.

**Figure 7:**
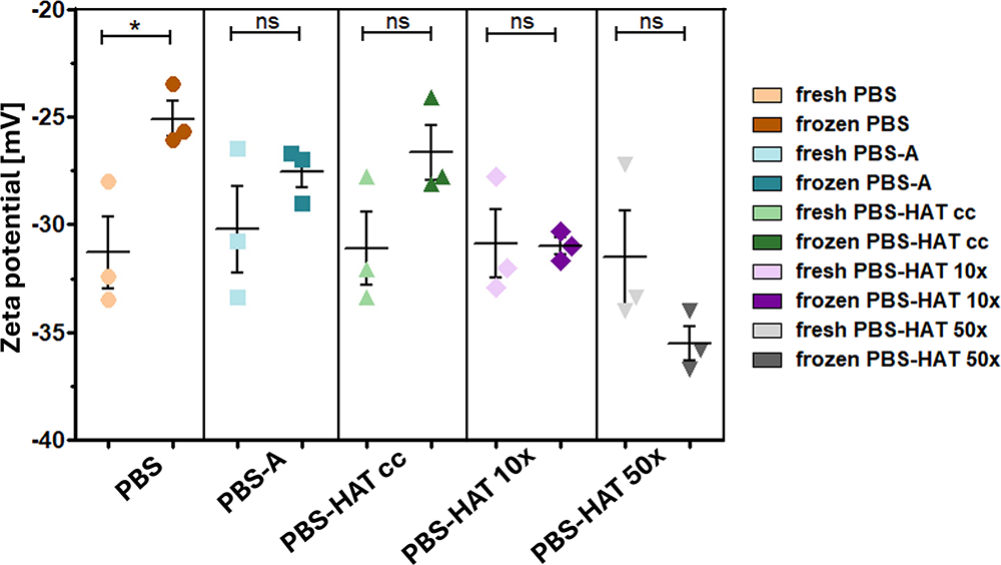
Zeta potential measurements of particles in fresh and thawed samples under various buffer conditions (PBS, PBS-A, PBS-HAT, PBS-HAT 10×, and PBS-HAT 50×). Data are presented as mean ± SD. One-way ANOVA followed by Bonferroni’s multiple comparison test were used for statistical analysis.

## 4 Discussion

The cryopreservation of EVs remains a major challenge in translational applications due to the structural fragility and complex composition of EVs. In this study, we aimed to systematically investigate how buffer compositions and vesicle concentration impact the preservation of REVs during freezing. Using several analytical techniques, we aimed to obtain a comprehensive picture of how freezing affected REV composition, particle recovery, size distribution, and structural integrity.

Our results show that buffer composition plays a crucial role in protecting membrane integrity and preserving EV structure during the freeze–thaw cycle. Consistent with previous studies,^4,12,34^ we found that cryopreservation in PBS, despite its widespread use in EV studies, leads to significant losses in protein and lipid content, hemoglobin retention, and particle concentration. Both the protein and lipid contents in EV-rich fractions decreased significantly, though to differing extents (49% and 78.7% recovery, respectively), aligning with previous findings.^4,35^ A new insight from our study is that the hemoglobin content of REVs is particularly sensitive to cryopreservation-related stress, with a recovery rate of only 23% when stored in PBS. This is especially important given that hemoglobin strongly influences the optical properties of REVs. The P/L quality marker calculated from colorimetric assays revealed a clear decline, which was further validated by the spectroscopic P/L ratios determined by infrared spectroscopy.

The changes in particle concentration were assessed by MRPS, which is unaffected by optical changes and FCM and can detect structural or biochemical alterations in EVs due to its greater sensitivity to changes in sample composition and refractive index. Significant particle loss was found by MRPS and was supported by the FCM results in the case of PBS-frozen samples. Additionally, scattering cross-section distributions showed shifts toward lower values, which most likely indicate a decreased refractive index caused by protein loss. However, these findings conflict with the results of de Oliveira Junior et al., who showed that despite differences in size and composition, RBC-derived microvesicle populations had a similar effective refractive index of 1.42.^32^ After the REVs were frozen in PBS, fluorescence labeling with the CD235a-PE antibody revealed the emergence of subpopulations characterized by CD235a positivity but reduced scattering cross-section, suggesting compositional changes. These results demonstrate how crucial buffer optimization is to maintain the structural integrity and reliable optical detectability of REVs.

Interestingly, the addition of HSA had no detectable protective effect, as no improvement in vesicle stability or recovery was seen during cryopreservation. However, the PBS-HAT buffer, which contains HSA, trehalose, and HEPES, provided much better protection, preserving important biochemical and biophysical properties of REVs, such as the protein-to-lipid ratio, hemoglobin content, size distribution, and scattering cross-section distribution. The buffer’s effectiveness may result from the combined action of its components: trehalose stabilizes lipid bilayers and prevents membrane fusion during freezing, HEPES maintains pH stability under freezing stress, and HSA can reduce vesicle aggregation and surface adsorption. The results were in line with those of Görgens et al., who also identified PBS-HAT as the optimal cryopreservation buffer for cell-culture-derived EVs,^4^ suggesting that this strategy may be broadly applicable across different EV subtypes.

Our study further reveals that the vesicle concentration at the time of freezing plays a significant role in REV recovery even when the optimal buffer composition is used. While concentrated PBS-HAT samples were well preserved, diluted samples (10× and 50×) showed notable particle losses despite maintained size and scattering characteristics of the remaining vesicles. This indicates that at lower concentrations, vesicles may be more prone to adsorption onto the SEC column or tube walls, as observed in a recent study;^36^ to aggregation; or to damage caused by the mechanical stresses of freezing and thawing. This aspect needs more research because it has been relatively understudied in the EV field.

Results of zeta potential measurements indicated that the surface charge of REVs are relatively stable across different storage conditions. However, a modest increase in the zeta potential was observed in PBS-frozen samples. This might be a result of aggregation or changes in the EV surface characteristics. There are conflicting results in the literature; although some studies have found that freezing alters zeta potential,^11^ others have found little to no effect,^12^ which is in line with our observation.

Importantly, previous studies have demonstrated that EV’s biological activity is closely linked to key biophysical and biochemical parameters, including membrane integrity, molecular composition, size distribution, and particle concentration. Accordingly, changes in EV composition and surface protein content, as shown by lower P/L ratios and the appearance of new subpopulations following cryopreservation in PBS and PBS-A, are expected to reduce bioactivity and cellular uptake.^5,30^ Given the dose-dependent nature of EV bioactivity,^4^ the particle loss observed after freezing in PBS or PBS-A is likely to reduce the effective EV dose and thereby impact functional outcomes. Furthermore, in REVs, hemoglobin retention is also key determinant of bioactivity, as REV-associated hemoglobin scavenges nitric oxide (NO) and modulates vasoconstriction.^37^ Our data show that storage in PBS and PBS-A results in a pronounced hemoglobin loss. In contrast, no significant changes were observed after storage in PBS-HAT in molecular composition, particle concentration, and hemoglobin retention, suggesting a maintained functional potential. These findings highlight the importance of storage conditions for maintaining EV bioactivity and warrant further investigation in future work.

It is worth noting that the 1 month storage period used in our study provided insights into short- to midterm stability but did not fully represent long-term preservation conditions. Therefore, longer storage studies up to several years will be needed to confirm PBS-HAT’s long-term suitability for cryopreservation. Furthermore, although we measured physical and biochemical parameters, functional analyses like cellular uptake were beyond the scope of this study and should be addressed in future work to fully validate the preservation of biological activity and PBS-HAT’s potential for clinical and research applications involving EVs.

## 5 Conclusions

As REVs continue to draw interest for their potential therapeutic and diagnostic purposes, the development of reliable cryopreservation protocols has become important. This study aimed to provide a practical framework for improving REV handling and storage for both research and potential clinical use. To determine the optimal storage conditions, the effects of buffer composition and vesicle concentration on the structural and biochemical integrity of REVs were systematically investigated during freezing. PBS-HAT, a buffer containing HSA, trehalose, and HEPES, performed significantly better than standard PBS in terms of preserving the particle integrity, hemoglobin content, and optical characteristics. Our results also demonstrate that the initial concentration of vesicles at the time of freezing significantly affects particle recovery since diluted samples were more susceptible to loss and damage. These observations emphasize how important it is to improve cryopreservation protocols. While our results demonstrate short- to midterm stability, further studies are needed to evaluate the effects of long-term storage and post-thaw biological functionality.
